# New Insights on Different Response of MDMA-Elicited Serotonin Syndrome to Systemic and Intracranial Administrations in the Rat Brain

**DOI:** 10.1371/journal.pone.0155551

**Published:** 2016-05-18

**Authors:** Ibrahim M. Shokry, John J. Callanan, John Sousa, Rui Tao

**Affiliations:** 1 Ross University School of Veterinary Medicine, St. Kitts, West Indies; 2 Charles E. Schmidt College of Medicine, Florida Atlantic University, Boca Raton, Florida, United States of America; The University of Texas at Austin, UNITED STATES

## Abstract

In spite of the fact that systemic administration of MDMA elicits serotonin syndrome, direct intracranial administration fails to reproduce the effect. To reconcile these findings, it has been suggested that the cause of serotonin syndrome is attributed mainly to MDMA hepatic metabolites, and less likely to MDMA itself. Recently, however, this explanation has been challenged, and alternative hypotheses need to be explored. Here, we tested the hypothesis that serotonin syndrome is the result of excessive 5HT simultaneously in many brain areas, while MDMA administered intracranially fails to cause serotonin syndrome because it produces only a localized effect at the delivery site and not to other parts of the brain. This hypothesis was examined using adult male Sprague Dawley rats by comparing 5HT responses in the right and left hemispheric frontal cortices, right and left hemispheric diencephalons, and medullar raphe nucleus. Occurrence of serotonin syndrome was confirmed by measuring change in body temperature. Administration routes included intraperitoneal (IP), intracerebroventricular (ICV) and reverse microdialysis. First, we found that IP administration caused excessive 5HT in all five sites investigated and induced hypothermia, suggesting the development of the serotonin syndrome. In contrast, ICV and reverse microdialysis caused excessive 5HT only in regions of delivery sites without changes in body-core temperature, suggesting the absence of the syndrome. Next, chemical dyes were used to trace differences in distribution and diffusion patterns between administration routes. After systemic administration, the dyes were found to be evenly distributed in the brain. However, the dyes administered through ICV or reverse microdialysis injection still remained in the delivery sites, poorly diffusing to the brain. In conclusion, intracranial MDMA administration in one area has no or little effect on other areas, which must be considered a plausible reason for the difference in MDMA-elicited serotonin syndrome between systemic and intracranial administrations.

## Introduction

Systemic administration of MDMA elicits excessive increases in extracellular serotonin (5-hydroxytryptamine; 5HT) in many brain areas [1,2], causing a wide spectrum of symptoms, including locomotion problems, anxiety and hyperthermia, collectively called serotonin syndrome [3]. These effects may be attributed to MDMA molecules that can be taken into serotonergic axon terminals, which leads to the expulsion of 5HT from storage vesicles into the extracellular space [4]. When 5HT exceeds 10-fold above baseline, it becomes toxic to postsynaptic neurons [5].

Intracranial administration is believed to be a powerful method to investigate the direct central nervous system effects of drugs. Schmidt and Tayler (1988) reported that dosages of MDMA, up to 300 μg, administered into one of the lateral ventricles (LV) failed to cause behavioral serotonin syndrome, changes in cortical or striatal tryptophan hydroxylase activity or cortical 5HT levels [6]. Similar observations were repeatedly reported by Molliver et al. (1986), Paris and Cunningham (1990), Callaway and Geyer (1992), and Esteban et al. (2001) [7–10]. Thus, it appears that intracranial administration failed to reproduce the toxic effect of this drug. In contrast, all systemic routes of administration, either subcutaneously [11], intraperitoneally [12] or intravenously [1] consistently produced the toxic effect. Not much is known as to the cause of the difference between intracranial and systemic routes. One widely acceptable explanation is that MDMA is not toxic unless metabolized through the liver [13–15]. Recently, however, this explanation has been challenged, demonstrating that MDMA metabolites do not penetrate the blood-brain barrier. Furthermore, the synthesized metabolites directly being administered into the brain do not cause neurotoxicity [16]. Therefore, alternative hypotheses need to be explored.

The goal of the present study was to investigate mechanisms that could be responsible for the different effects on serotonin syndrome between systemic and intracranial MDMA administrations. It is known that serotonin syndrome is a triad of mental status changes, neuromuscular hyperactivity and autonomic dysfunction [17,18]. Considering such a diversity of symptoms, it is reasonable to believe that many brain areas are simultaneously involved in the syndrome processing. In mammalians, the prefrontal cortex (FCx) is one of the critical brain areas that are involved in controlling mental status [19] and neuromuscular activity [20]; diencephalons (DEc) and medullar raphe (MRp) are in the pathway controlling autonomic activity [21]. However, although the role of individual areas in mediating specific symptoms of serotonin syndrome is not established [22], it is clear that serotonin syndrome is a result of excessive 5HT simultaneously in many brain areas, but unlikely a single area. In the present studies, we tested the hypothesis that MDMA administered intracranially causes a localized effect only at the delivery site, but not a widespread effect in several areas that are simultaneously involved in the syndrome processing. To test this hypothesis, we utilized the microdialysis probes to examine extracellular 5HT in the FCx, DEc and MRp in response to systemic and intracranial administration of MDMA. In addition, we compared the 5HT responses in the right hemisphere with that in the left hemisphere. Furthermore, we tested that drug distribution is different between routes of administration, predicting that the intracranial route will have poor diffusion from the administration site to the interstitial fluid (ISF) [23] within the brain. This hypothesis was indirectly examined by utilizing chemical dyes, fast green and DiI. Our results support a new hypothesis that poor diffusion of MDMA to the ISF, and inability to reach distant areas that are critical for the syndrome processing is likely the cause of failure of serotonin syndrome elicited by MDMA following intracranial injection.

## Materials and Methods

### Animals

Adult male Sprague–Dawley rats weighing 250–275 g were purchased from Charles River Laboratories (Raleigh, NC, USA) and group-housed (two to three per cage) in a temperature (22 ±1°C)- and humidity (40–70%)-controlled facility on a 12-h light/dark cycle (lights on from 07:00–19:00). Food and water were available at all times. Animals were assigned to experiments when their body weight reached 300–350 g. All animal use procedures were in strict accordance with the National Institutes of Health Guide for the Care and Use of Laboratory Animals and approved by the Institutional Animal Care & Use Committee (IACUC) at Florida Atlantic University and Ross University School of Veterinary Medicine. Special care was taken to minimize animal discomfort during all procedures.

### Chemicals

Racemic MDMA [(±)-3,4-methylenedioxymethamphetamine hydrochloride] was kindly provided by the National Institute of Drug Abuse (Baltimore, MD, USA). DiI and fast green were purchased from AnaSpec Inc (Fremont, CA, USA) and Fisher Scientific (Fair Lawn, NJ, USA), respectively. MDMA and fast green were dissolved in 0.9% NaCl for systemic injection, in artificial cerebrospinal fluid (aCSF; 140 mM NaCl, 3.0 mM KCl, 1.5 mM CaCl_2_, 1.0 mM MgCl_2_, 0.25 mM NaH_2_PO_4_, and 1.0 mM Na_2_HPO_4_) for ICV administration. DiI was dissolved in DMSO for systemic and intracranial administration.

### Cannulas guide implantation

Animals were anesthetized by IP injection of xylazine (4 mg/kg) and ketamine (80 mg/kg), and mounted on a Kopf stereotaxic frame. Animal skulls were implanted with five cannulas (10 mm long 22 gauge stainless steel), unless stated otherwise. The coordinates were the following: frontal cortex (FCx) in the right and left hemispheres, AP +3.20 mm relative to bregma, ML ±0.70 mm to midline, and DV -2.0 mm relative to the skull surface; diencephalon (DEc) in the right and left hemispheres, AP -1.10 mm relative to bregma, ML ±0.90 mm to midline, and DV -2.0 mm relative to the skull surface; medullar raphe (MRp), AP -2.0 mm relative to lambda, ML 0 mm, and DV -2.0 mm to the skull surface. After implantation, cannulas were secured to the skull using dental cement, and further protected with obturators to prevent blockage. Experiments began after a 7-day recovery period.

In the animals for reverse microdialysis administration of MDMA, a single (targeting to MRp) or bilateral guide cannulas (to FCx or DEc) were implanted into the rat skull using the coordinates described above. In a negative control study, two cannulas were implanted in the right FCx. The coordinates for the first guide were AP +3.20 mm relative to bregma, ML 0.70 mm to midline, and DV -2.0 mm relative to the skull surface. The second guide at a distance of 2 mm lateral to the first probe: AP +3.20 mm relative to bregma, ML 2.70 mm to midline, and DV -2.0 mm relative to the skull surface.

In the animals for ICV injection, a single guide cannula was implanted to the right lateral ventricle (LV) at coordinates of AP -0.40 mm relative to bregma, ML 1.50 mm, and DV -2.0 mm to the skull surface. An obturator was plugged into the cannula to prevent blockage or leakage of the CSF.

### ICV administration of MDMA

The injector needle was a 13 mm stainless steel tubing (26 gauge) which was connected to polyethylene tubing which, in turn, was attached to a Hamilton syringe fitted on to an infusion pump. The needle was inserted into the right LV through the guide cannula while animals remained in a Raturn bowl (BASi, West Lafayette, IN, USA). A total 2 μL was infused for 10 min to the right LV at infusion rate of 0.2 μl/min. The injector needle was left in place for an additional 10 min. Upon completion of ICV administration, the cannula was immediately closed with an obturator to prevent CSF leakage.

### Microdialysis and reverse microdialysis administration

We constructed a microdialysis probe from 26-gauge stainless steel tubing and glass silica. The dialysis tubing was hollow nitrocellulose fiber (200 μm i.d., 13,000 MW cut-off; Spectrum Medical Industries, Los Angeles, CA, USA). The length of the exchange surface of dialysis membrane was 2.5 mm.

The evening before an experiment, animals were briefly anesthetized with 1–2% isoflurane. Aseptic dialysis probes were inserted through the guide cannulas. Dialysis probe shafts were measured, and the length adjusted to the brain targets using collars prepared from 21-gauge stainless steel tubing. The target coordinates for the tip of the probe were in the FCx in the right and left hemispheres, AP +3.20 mm relative to the bregma, ML ±0.7 mm relative to the midline and DV -5.5 mm relative to the skull surface. While the coordinates for the DEc were in the right and left hemispheres, AP -1.10 mm relative to the bregma, ML ±0.90 mm relative to the midline and DV -9.50 mm relative to the skull surface. The coordinates for the MRp were AP -2.00 mm relative to lambda, ML 0, and DV -10.5 mm relative to the skull surface. The probes were secured to the skull using dental cement. Rats were then placed in the Raturn bowl that allowed animals to move freely. Food and water were available *ad libitum*. The microdialysis probe-inlets were perfused overnight with aCSF at a flow rate of 1.0 μl/min.

The next day, samples were collected every 15 min at a flow rate of 1.0 μl/min (brain sites terminated from the study if the flow rate was lower than 1.0 μl/min). After 4 consecutive baselines, MDMA was administered either through IP administration of 2 mg/kg, ICV administration of 5–100 μg or reverse microdialysis at concentrations of 1–30 μM for 30 min. Upon completion of microdialysis, animals were deeply anesthetized with ketamine (120 mg/kg, IP) in combination of xylyzine (4 mg/kg, IP). Probes were infused with 2% fast green for 5 min. Brains were removed and frozen at -80°C. Probe placements were verified by viewing tracks in comparison with the coronal brain slices from the Paxinos and Watson craniometric rat brain atlas [24]. Seven probes in this study were off-targets and removed from data analysis.

### Serotonin (5HT) assay

We analyzed the samples using a high-performance liquid chromatography (HPLC) with electrochemical detection (HPLC-EC; HTEC-500, Eicom, Japan). 5HT was separated through a mobile phase (0.1 M phosphate buffer at pH 6.0, 500 mg/L 1-decanesulfonic acid, 50 mg/L EDTA, and 1.0% methanol) at a rate of 0.50 ml/min. The detection limit for 5-HT was approximately 0.05 pg per sample based on a signal-to-noise ratio of 3:1. The mean of four consecutive measurements was obtained as a baseline, immediately before drug or vehicle injection. Changes in the 5HT overflow were expressed as a fold increase above baseline. Data were expressed as mean ± s.e.m, indicating the time course of the 5HT overflow, both before and after injection.

### Core temperature (*T*_cor_) measurements

Changes in *T*_cor_ were used to estimate the adverse effect relevant to autonomic activity induced by MDMA. Rats were allowed to acclimatize to the temperature-controlled measurement chamber (set at 22 ±1°C) for at least a 2 h period prior to obtaining body temperature measurements. A 7-mm flexible thermoprobe was inserted into the rectum, and *T*_cor_ displayed on a digital monitor. To minimize physical disturbance, the thermoprobe extension cord was secured on rat tail and thus *T*_cor_ was continuously recorded at 5 min intervals throughout the experiment. Four consecutive measurements, immediately before drug or vehicle injection, were averaged as a baseline value. Changes in *T*_cor_ from the baseline were used in data analysis. Data were expressed as mean ± s.e.m, indicating the time course of *T*_cor_ changes, both before and after injection.

### Protocol for DiI and fast green tests

In this set of experiments, all animals were deeply anesthetized with xylazine (4 mg/kg, IP) and ketamine (120 mg/kg, IP). For the IV administration, 3 mg of fast green was dissolved in 0.15 ml of 0.9% NaCl or 0.3 mg DiI in 0.15 ml of DMSO. Injection took place in the tail vein using a scalp vein set (27-gauge; Exelint International Co., LA, USA). The brain was removed in 30 min. However, fast green in the FCx was not observed with naked-eye or microphotographic examination (data not shown). For the reverse microdialysis administration, fast green was dissolved into aCSF at a concentration of 24 mM, and DiI dissolved in DMSO at a concentration of 5 mM. Infusion time was 30 min at a flow rate of 1 μL/min. For the ICV administration, 40 μg of fast green dissolved in 2 μL of aCSF or 4 μg of DiI in 2 μL of DMSO was delivered through a right LV cannula at a flow rate of 0.2 μL/min. After the ICV administration, the cannula remained in the right LV for an additional 10 min. The brain was removed in 30 min from the skull and briefly frozen in a methanol/dry ice bath and stored at -80°C until sectioning. The brain was then embedded within OCT, mounted on a chuck in a cryostat (Leica CM-1850, Nussloch, Germany). In the fast green study, coronal sections through the brain were photographed on the chuck using a digital camera (Nikon D7000). In the DiI study, the brain was sliced into 40 μm of coronal sections. Sections were mounted on a glass slide and examined using a Provis AX70 microscope equipped with CellSens software (Olympus, Tokyo, Japan).

### Statistical analysis

Statistical analysis was conducted with 5HT and *T*_cor_ data in which independent variables are drugs and vehicle. To bring the variables into proportion with one another, the 5HT data were normalized to fold increases from baseline values. There are 6 dependable variables identified (15 min, 30 min, 45 min, 60 min, 75 min and 90 min). For *T*_cor_ studies, data were normalized to changes from baseline *T*_cor_ prior administration. Dependable variables consist of 18 time points of data collections (5 min, 10 min, 15 min, 20 min, 25 min, 30 min, 35 min, 40 min, 45 min, 50 min, 55 min, 60 min, 65 min, 70 min, 75 min, 80 min, 85 min, and 90 min). Thus, the one-way analysis of variance (ANOVA) with repeated measures was used to determine whether there were any significant differences between vehicle and MDMA administration in a given brain area. If significant difference between drug treatment and vehicle was found, further statistical analysis was carried out using a *post-hoc* test in determining the significance of respective time points. It should be noted that the sample size between groups may differ from each other because of sample loss due to probe misplacement or lower flow rate (<1 μl/min). Despite this, a sample size in a given brain site should have at least 4 cases, and the loss in some occasions would not affect data analysis. Since the Scheffe approach is highly flexible, and can be used for both equal and unequal sample sizes, it was thus employed for the post-hoc test. The level for statistical significance was set at 0.05.

## Results

### Effect of MDMA-administration routes

**Fig 1:**
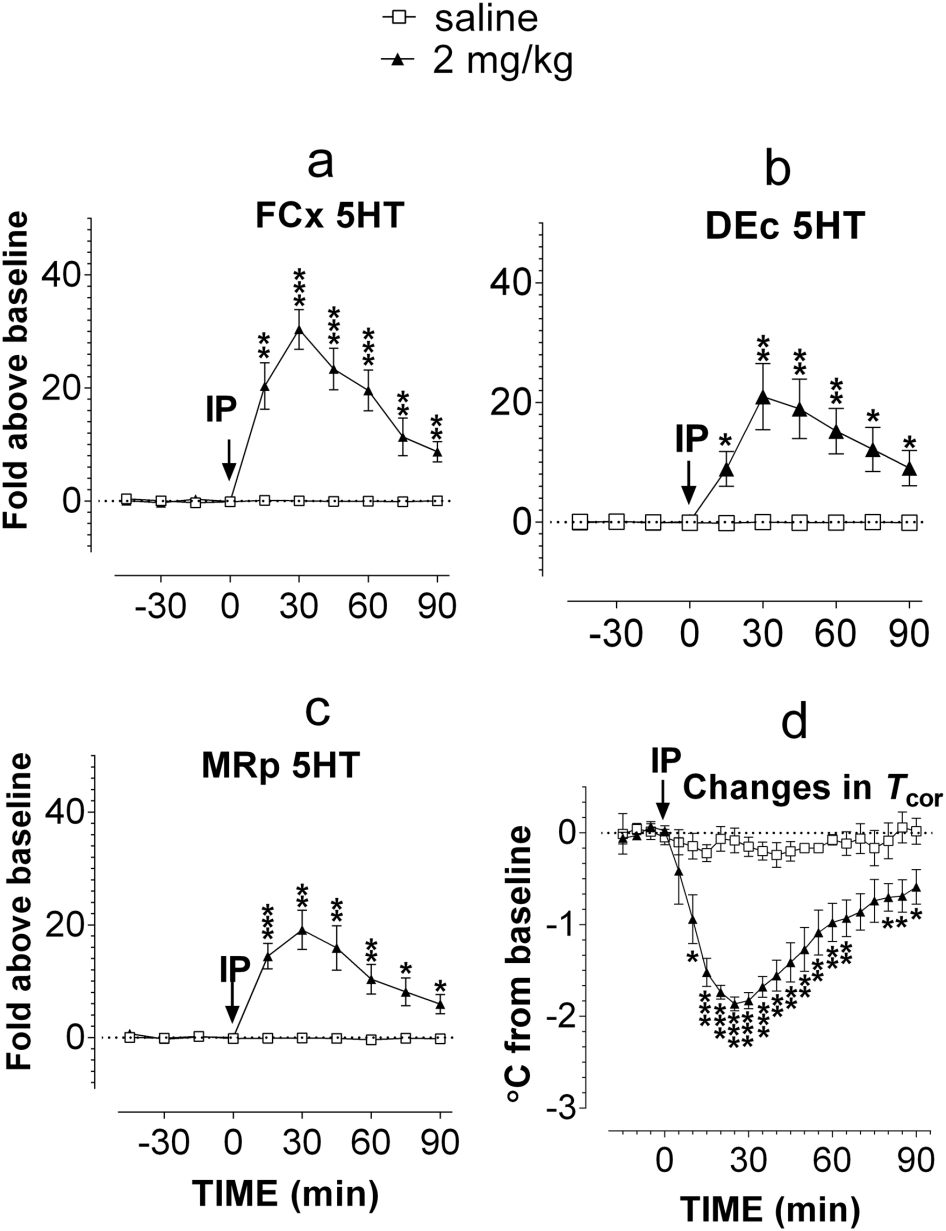
Relationship between systemic injection of MDMA and occurrence of serotonin syndrome in male Sprague-Dawley rats. The route of systemic administration was IP. Serotonin syndrome was determined by measuring extracellular 5HT in the FCx, DEc and MRp, and further verified by measuring changes in body-core temperature (*T*_cor_). Arrows indicate time of IP injection at a dose of 2 mg/kg MDMA. Data are expressed as mean ± s.e.m. Extracellular 5HT is excessively elevated (>10-fold) in a, the FCx (n = 5 rats/group); b, the DEc (n = 5 rats/group); c, the MRp (n = 4–5 rats/group). d, *T*_cor_ becomes hypothermia (n = 5 rats/group). * P <0.05 *vs*. saline control, determined by Scheffe’s test followed by one-way repeat measures ANOVA.

First, 2 mg/kg MDMA was injected intraperitoneally (IP) to rats while 5HT was measured simultaneously in five regions: the right and left hemispheric FCx, the right and left hemispheric DEc, and MRp. Since there was no difference in 5HT response between two hemispheric regions, two FCx or DEc data were merged to reduce numbers of animals used in the study. In this study, five animals were used for testing MDMA and another five rats for saline injection. A probe to the MRp was off-target and thus omitted from the data analysis. As compared to saline controls at the respective microdialysis sites, MDMA administration caused a significant 5HT elevation in the FCx (Fig 1a; F_(1,8)_ = 36.429, P = 0.0003), DEc (Fig 1b; F_(1,8)_ = 14.046, P = 0.0056), and MRp (Fig 1c; F_(1,7)_ = 18.468, P = 0.0036). The maximum elevation was ~30 fold in the FCx, ~20 fold in the DEc and MRp. In a separate experiment, IP administration of 2 mg/kg MDMA resulted in a significant reduction in body-core temperature (*T*_cor_) (Fig 1d; F_(1,8)_ = 24.875, P = 0.0011).

**Fig 2:**
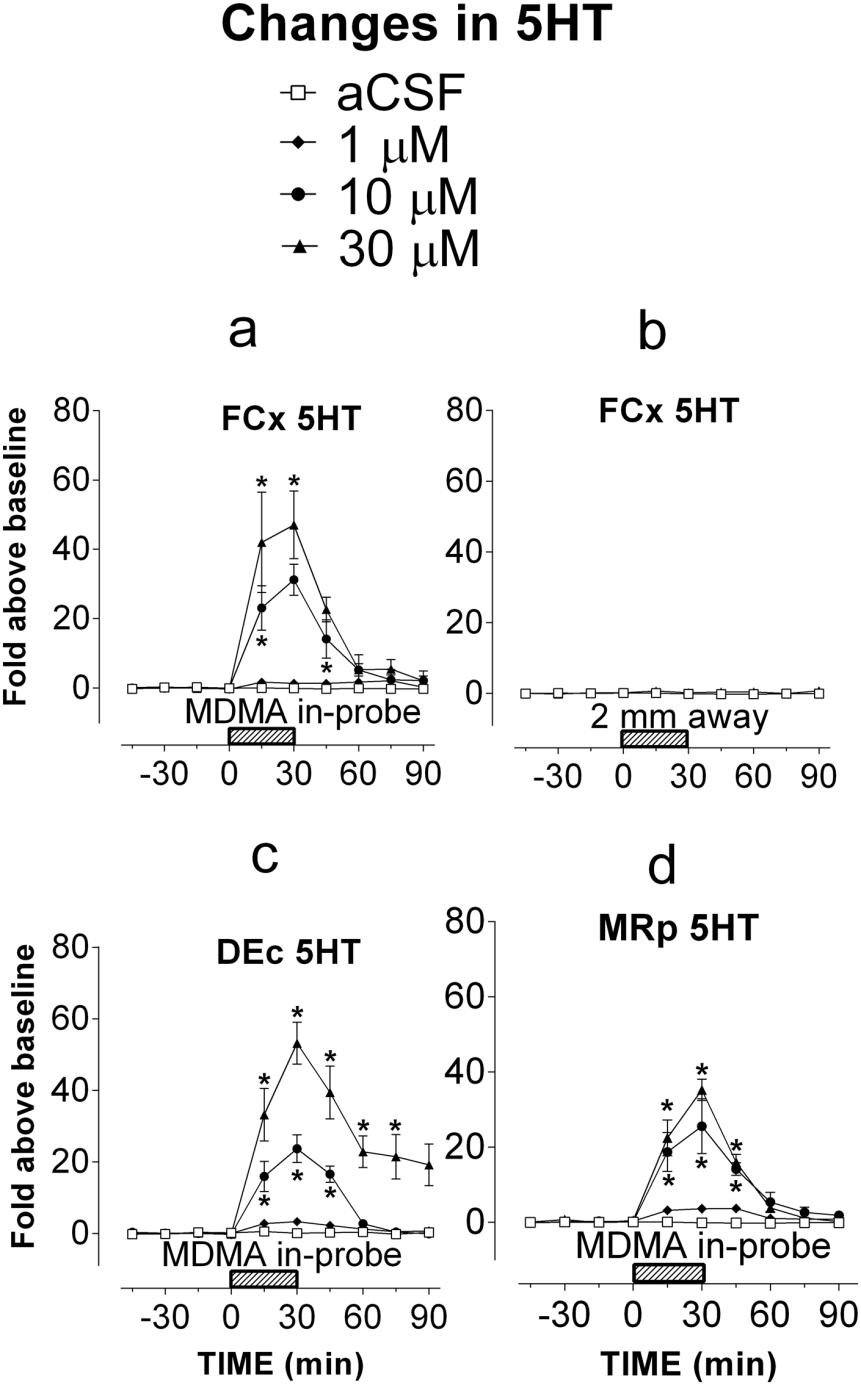
Relationship between intracranial administration of MDMA and occurrence of serotonin syndrome in male Sprague-Dawley rats. The route of intracranial administration was reverse microdialysis. Serotonin syndrome was determined by measuring extracellular 5HT in the delivery sites (FCx, DEc and MRp), or a site at 2 mm away from the delivery site. Hatched horizontal bars indicate the time period of reverse microdialysis at MDMA concentrations of 0 (aCSF; □), 1 μM (◆), 10 μM (●) or 30 μM (▲). Data are expressed as mean ± s.e.m. a, Reverse microdialysis in the FCx caused concentration-dependent elevation at the delivery site (n = 4–6 rats/group). b, Reverse microdialysis of 30 μM MDMA in the FCx had no effect on extracellular 5HT in the site at 2 mm away from the delivery site (see Materials and Method section). c, Reverse microdialysis in the DEc caused MDMA concentration-dependent elevation at the delivery site (n = 6–8 rats/group). d, Reverse microdialysis in the MRp caused MDMA concentration-dependent elevation at the delivery site (n = 4–6 rats/group). * P <0.05 *vs*. saline control, determined by Scheffe’s test followed by one-way repeat measures ANOVA.

**Fig 3:**
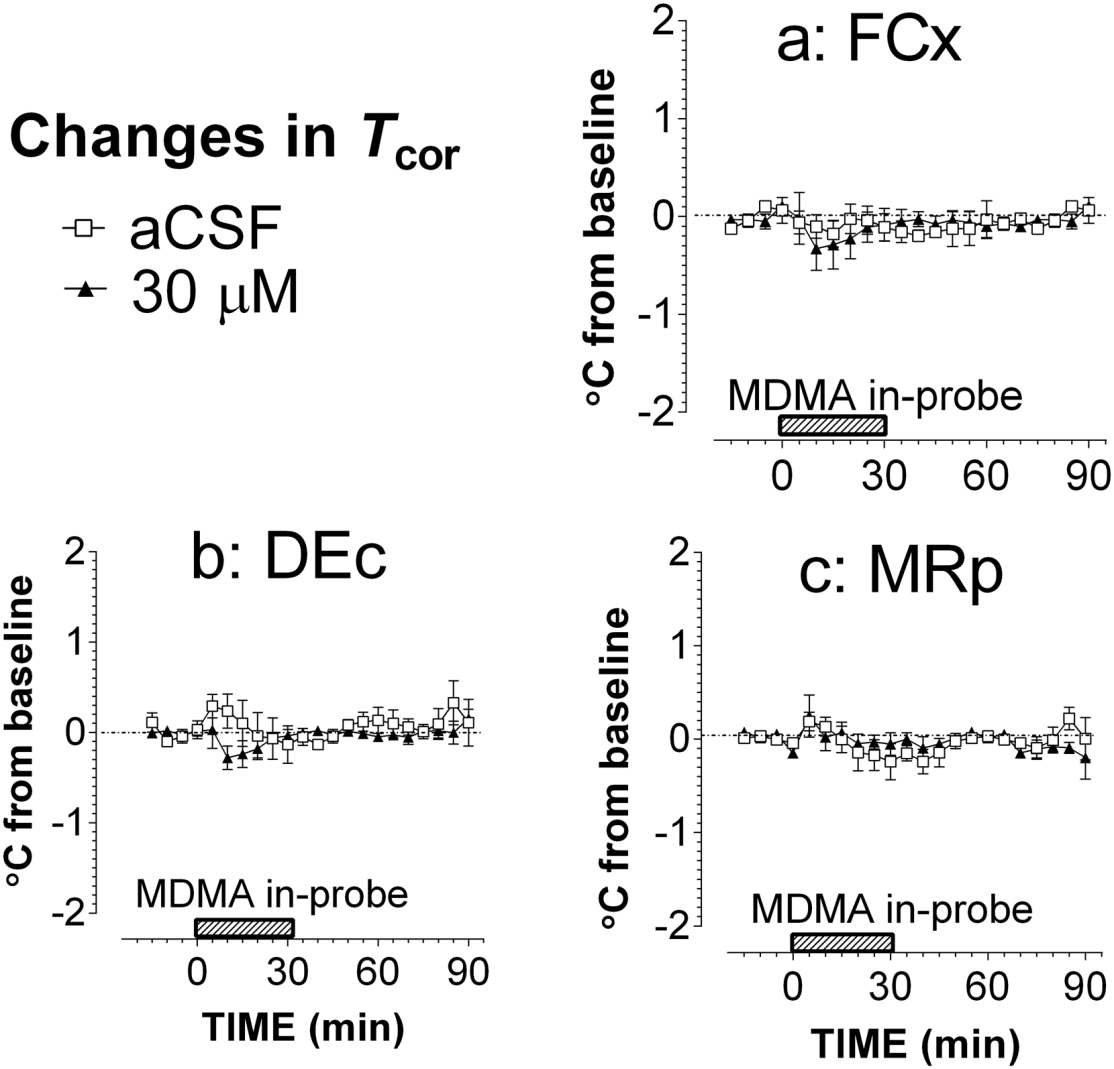
Relationship between intracranial administration of MDMA and occurrence of serotonin syndrome in male Sprague-Dawley rats. The route of intracranial administration was reverse microdialysis. Serotonin syndrome was determined by measuring changes in body-core temperature (*T*_cor_). Hatched horizontal bars indicate the time period of aCSF (□), or 30 μM MDMA infusion (▲) in the delivery site. Data are expressed as mean ± s.e.m. a, Bilateral reverse microdialysis in the FCx had no effect on *T*_cor_ (n = 5 rats/group). b, Bilateral reverse microdialysis in the DEc had no effect on *T*_cor_ (n = 5–6 rats/group). c, Singular reverse microdialysis in the MRp had no effect on *T*_cor_ (n = 5–6 rats/group).

Next, bilateral probes were implanted in the right and left hemispheric FCx or DEc, or a singular probe to MRp of rats. We found that 5HT changes in bilateral probes were similar, and thus bilateral data merged to reduce numbers of animals used in the study. Except for the DEc group, each MDMA concentration (1, 10, and 30 μM) added to the infusion medium was examined with 5 different rats through microdialysis probes while 5HT was determined at the same probes. In the DEc test, each concentration was examined with seven different animals. Of those, two probes were omitted from data analysis because of a low flow rate (<1 μl/min) in the probes. MDMA caused a concentration-dependent increase in the FCx (Fig 2a; F_(3,16)_ = 49.98, P <0.0001), DEc (Fig 2c; F_(3,22)_ = 31.474, P <0.0001) and MRp (Fig 2d; F_(3,16)_ = 32.799, P <0.0001). The maximum increase was ~45 fold in the FCx, 40 fold in the DEc and 38 fold in the MRp in response to 30 μM MDMA. To determine the extent of MDMA diffusion, we conducted a negative study by implanting two probes in the right FCx hemisphere but being separated at a distance of 2 mm of each other. Changes in extracellular 5HT in one probe were measured while aCSF (N = 6) or MDMA at a concentration of 30 μM (N = 5) was delivered in another probe. As shown in Fig 2b, 5HT was not significantly altered 2 mm away from the MDMA delivery site (F_(1,9)_ = 4.127, P = 0.0728). Next, *T*_cor_ of rats was determined while 30 μM of MDMA was infused bilaterally into the FCx (Fig 3a) or DEc (Fig 3b), or singularly to MRp (Fig 3c). MDMA directly infused into either of these regions did not have an effect on *T*_cor_ (FCx, F_(1,8)_ = 0.054, P = 0.8228; DEc, F_(1,9)_ = 0.0005, P = 0.9455; MRp, F_(1,9)_ = 0.348, P = 0.5696).

**Fig 4:**
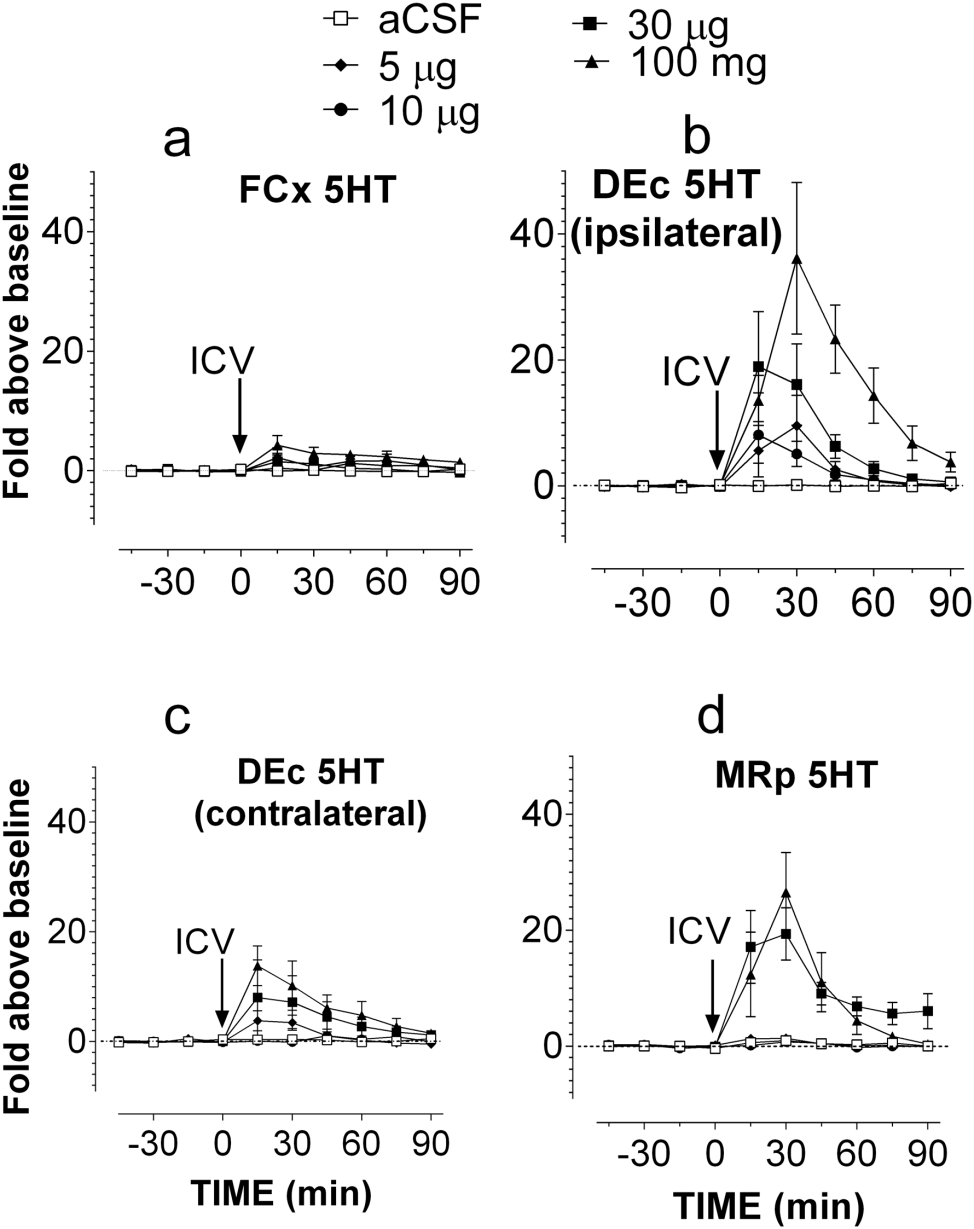
Relationship between intracranial injection of MDMA and occurrence of serotonin syndrome in male Sprague-Dawley rats. The route of intracranial administration was ICV. Serotonin syndrome was determined by measuring extracellular 5HT in the FCx, DEc and MRp. Arrows indicate the time of ICV administration at doses of 0 (aCSF; □), 5 μg (◆), 10 μg (●), 30 μg (■) or 100 μg (▲). Data are expressed as mean ± s.e.m. a, FCx (n = 5–6 rats/group). b, Ipsilateral DEc (n = 4–6 rats/group). c, Contralateral DEc (n = 5–6 rats/group). d, MRp (n = 4–6 rats/group). For the purpose of clarification, symbols indicating statistical significance were omitted from the graphs.

**Fig 5:**
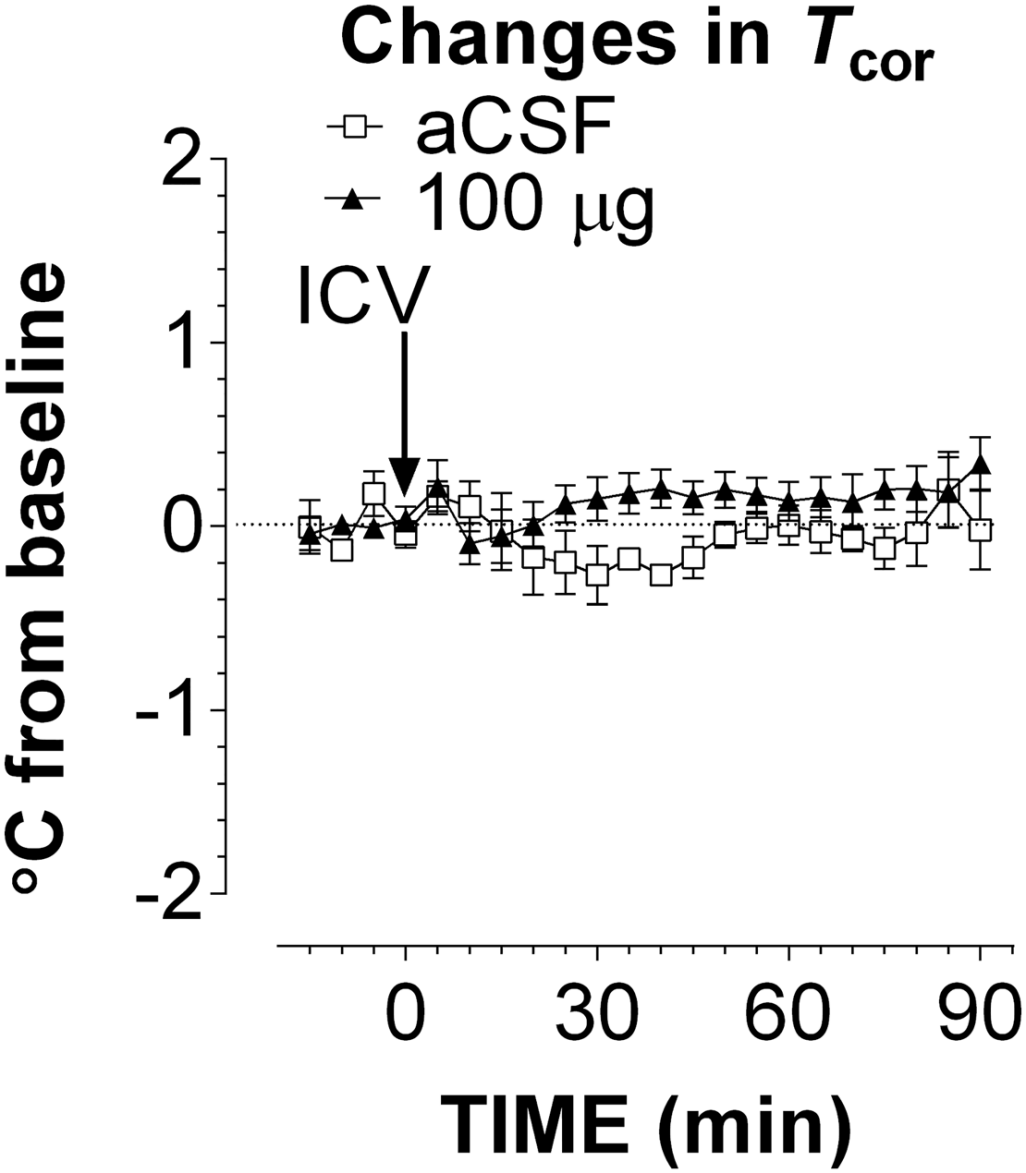
Relationship between intracranial injection of MDMA and occurrence of serotonin syndrome in male Sprague-Dawley rats. The route of intracranial administration was ICV. Serotonin syndrome was determined by measuring changes in *T*_cor_. Arrows indicate the time of ICV administration of aCSF (□) or 100 μg (▲). Data are expressed as mean ± s.e.m (n = 5 rats/group). ICV administration of 100 μg MDMA had no effect on *T*_cor_.

Lastly, MDMA at doses of 5, 10, 30 and 100 μg was delivered using ICV administration while 5HT was determined simultaneously in five regions: two FCx (the right and left hemispheres), two DEc (the right and left hemispheres), and one MRp (midline). Changes in 5HT were not different in the two hemispheric FCx. Therefore, data from two FCx sites were merged. Compared to aCSF control, ICV administration of 5–100 μg MDMA caused significant increases of 5HT in the FCx (Fig 4a; F_(4,23)_ = 4.007, P = 0.0131). However, these increases were less than 10-fold, which is lower than the threshold for serotonin syndrome. Interestingly, two DEc sites showed remarkable differences in response to ICV injection, and thus were analyzed separately. 5HT increases in the left hemispheric DEc (contralateral to the ICV injection site) were relatively small (Fig 4c). In contrast, there were significant increases in the right hemispheric DEc (ipsilateral to the ICV injection site) (Fig 4b; F_(4,20)_ = 7.865, P = 0.0006). The maximum elevation was 36 ±12 fold after 100 μg. In the MRp study, two probes were omitted from the data analysis due to the probe misplacement. 5HT in the MRp was also significantly elevated in a dose-dependent manner (F_(4,19)_ = 16.252, P <0.0001). In this regard, the maximum elevation was ~26 ±7 fold in response to 100 μg. In separate experiments; we determined whether *T*_cor_ was altered following ICV administration of MDMA. As shown in Fig 5, MDMA at 100 μg had no effect on *T*_cor_ (F_(1,8)_ = 3.024, P = 0.1202).

### DiI and fast green tests

**Fig 6:**
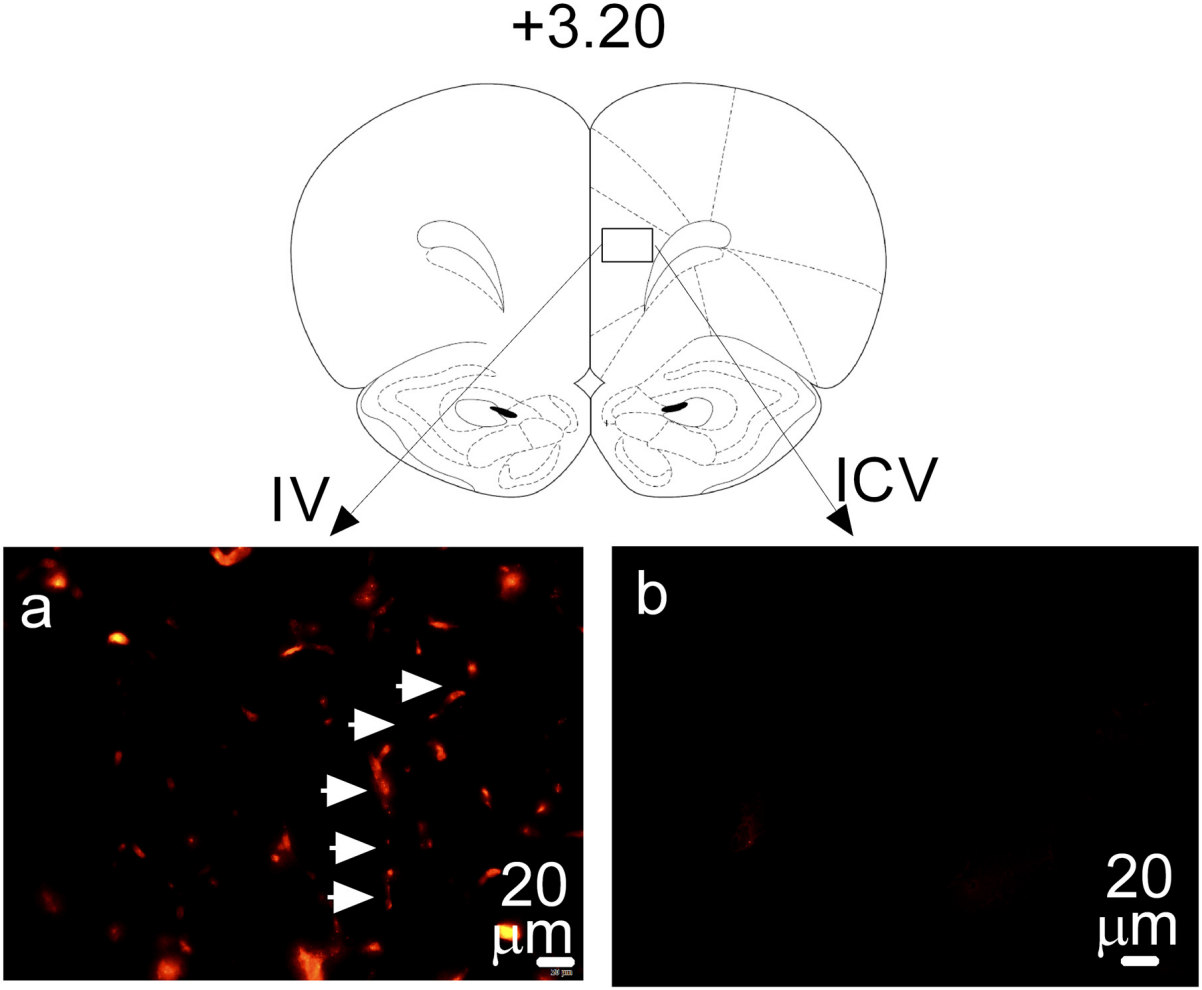
Relationship between routes of dye administration and distribution in the brain in male Sprague-Dawley rats. N = 4 rats/group. The dyes used in this test were DiI. FCx was used as a representative area for the brain. IV administration was used as a systemic administration, and ICV injection as an intracranial administration. Atlas coordinates in millimeters anterior to bregma, +3.20. Bars scale, 20 μm. a, IV administration at 300 μg/rat causes DiI distribution in the FCx capillaries (indicated by arrows). b, There is no DiI trace in the FCx following ICV administration of DiI (4 μg/head).

First, we compared the difference in DiI distribution between IV and ICV administration. IV administration resulted in a widespread distribution of DiI throughout the entire brain. FCx is used as a representative microphotograph as shown in Fig 6a. In contrast, there were no DiI traces in the FCx following ICV administration (Fig 6b).

**Fig 7:**
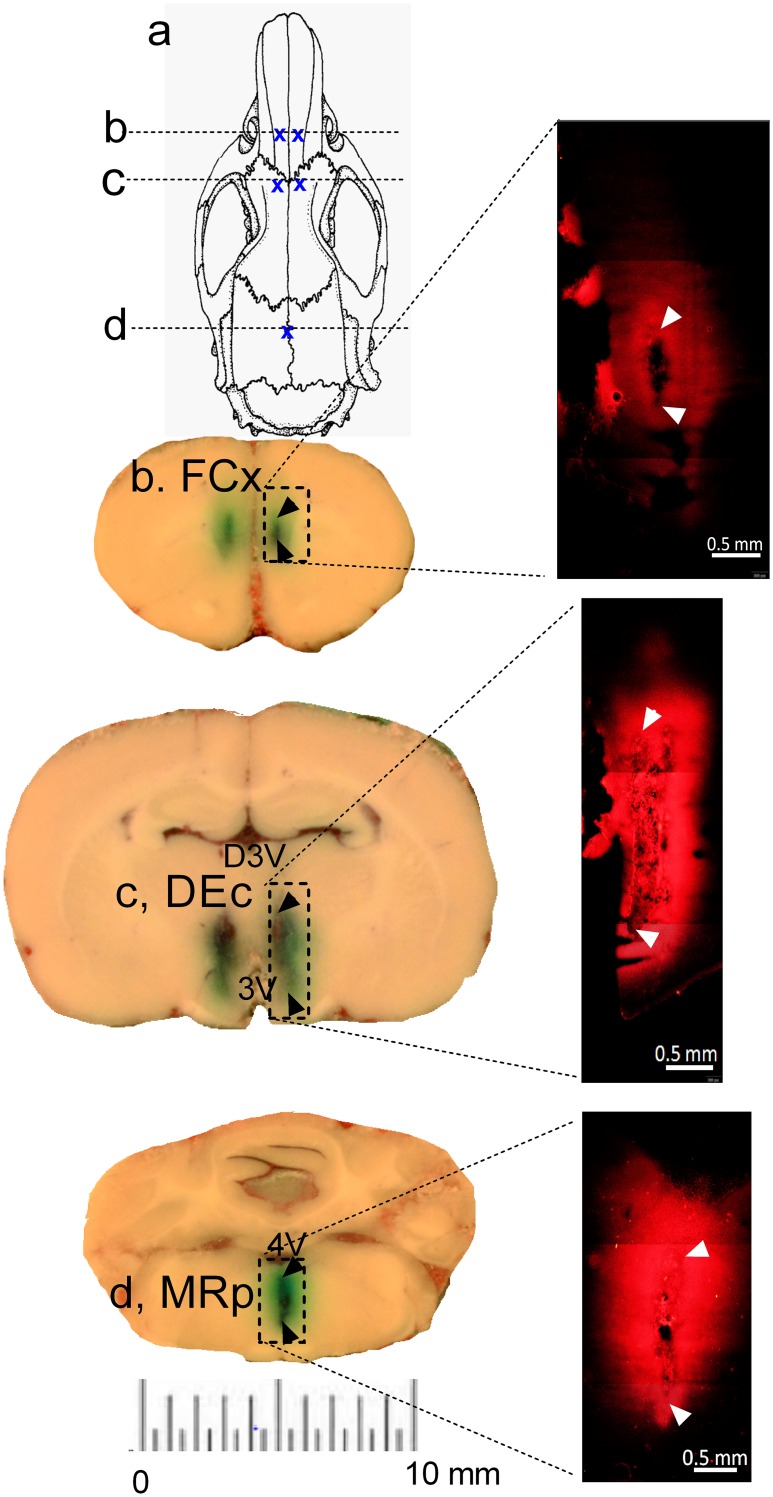
Relationship between intracranial administration and dye diffusion from the delivery site to the surrounding or distant tissue. The route of intracranial administration was reverse microdialysis. The dyes used in this test were fast green and DiI. N = 4 rats/group. a, A diagram illustrates a dorsal view of the location of guide cannulas and dash lines indicate where the brain was sliced. b, Bilateral reverse microdialysis administration of fast green in the FCx. Inset, DiI administration; c, Bilateral reverse microdialysis administration of fast green in the DEc. Inset, DiI; d, Singular reverse microdialysis administration of fast green in the MRp. Inset: DiI administration.

Next, we determined whether the five brain areas exhibited regional difference in drug distribution and diffusion. In this set of studies, fast green and DiI was delivered through reverse microdialysis administration. As shown in Fig 7, all five regions showed the same pattern of diffusion from microdialysis probes in the bilateral FCx, or bilateral DEc, or singular MRp. However, the distance of diffusion was less than 1 mm away from the probes (arrow heads indicative of probe tracks).

**Fig 8:**
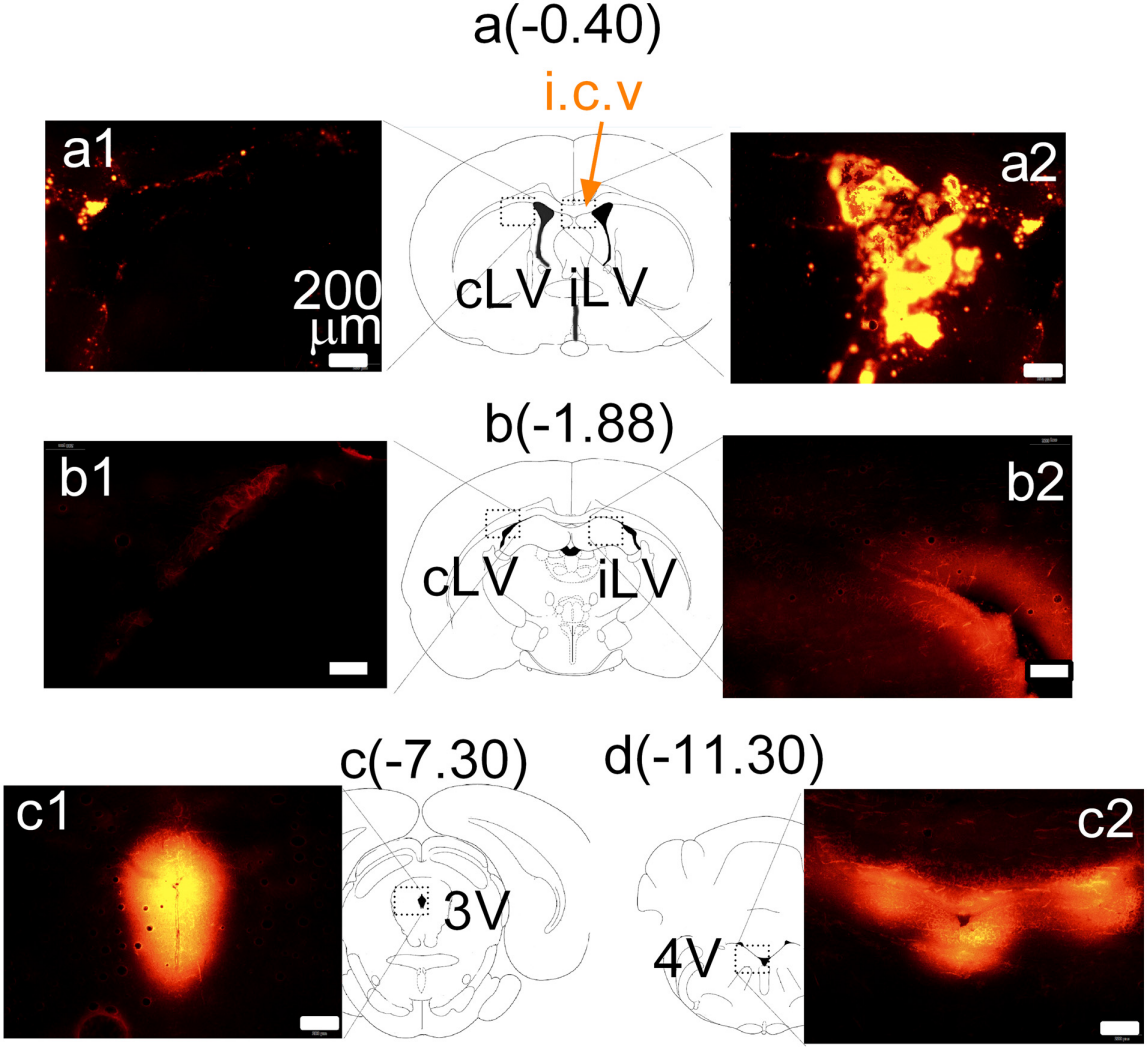
Relationship between intracranial administration and dye diffusion from the delivery site to the surrounding or distant tissue. The route of intracranial administration was ICV. The dyes used in this test were DiI. N = 4 rats. Atlas coordinates are in millimeters posterior to bregma at -0.40 (a), -1.88 (b), -7.30 (c), and -11.30 (d). Bars scale, 200 μm. DiI was injected at the right LV at coordinates of AP -0.40 mm (a2). DiI density was higher in the ipsilateral LV (iLV; a2 and b2) than that in the contralateral LV (cLV; a1 and b1). DiI fluorescence was found in the LV to 3V and 4V (c1 and c2). However, diffusion of DiI to the ISF is very poor, ~200 μm away from the CSF compartments.

**Fig 9:**
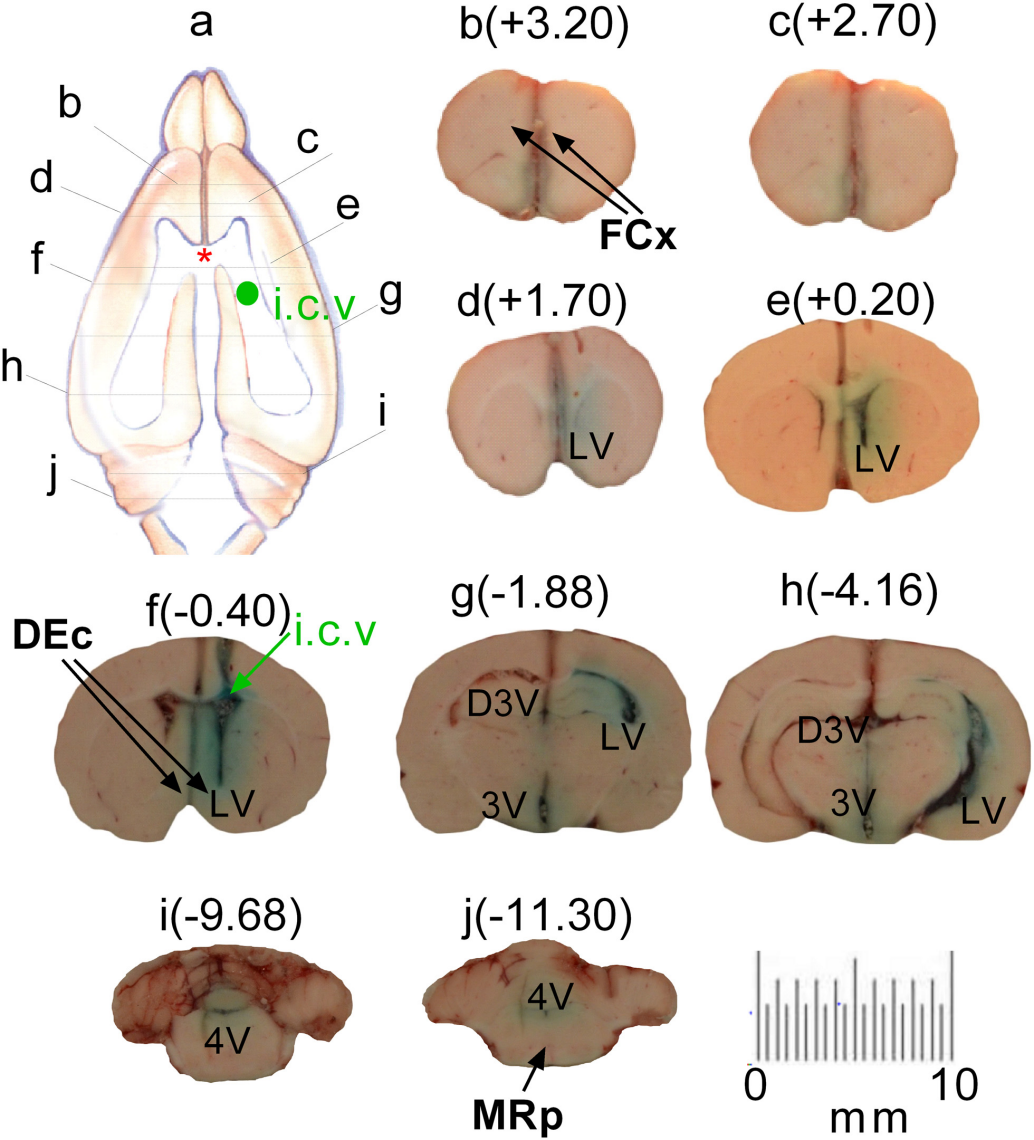
Relationship between intracranial administration and dye diffusion from the delivery site to the surrounding or distant tissue. The route of intracranial administration was ICV. The dyes used in this test were fast green. N = 5 rats. a, Diagram represents the atlas anterior or posterior to the bregma (*). Lines indicate where the section was cut. After ICV administration to the right LV (f), fast green was distributed within four ventricles, including the ipsilateral LV (d, e, f, g and h), 3V (g, h) and 4V (I, j), but poorly in the contralateral LV. Diffusion of fast green to the ISF was poor, ~1.5–2 mm away from the ventricle compartments. However, there was no fast green in the FCx (b, c).

Finally, we determined whether dyes administered in the CSF of brain cavities could diffuse to the ISF within the brain parenchyma. Dye movement in the CSF can be determined by observing the four ventricles (right and left LV, 3V and 4V), while movement in the ISF is estimated by observing the dye in the brain tissues. For a clear description, dye movement in the four ventricles was described as distribution, while dye exiting out of the ventricles as diffusion. As shown in Fig 8, DiI distributed widely in the four ventricles, but diffused poorly to the ISF compartment. Specifically, DiI administered at AP -0.40 was evident with a high density in the ipsilateral LV (Fig 8a2; labeled as iLV) whereas, only a few particles were found in the contralateral LV (cLV; Fig 8a1). DiI was observed in other parts of LV (Fig 8b). Moreover, DiI flowed to 3V (Fig 8c1) and 4V (Fig 8c2). In addition, DiI diffused out of the ventricles (Fig 8b2). The distance of diffusion was less than 200 μm away from the ventricle compartment. In a separate experiment, we studied distribution and diffusion of fast green 30 min after ICV administration. Fig 9 showed that fast green distributed broadly into the iLV, 3V and 4V but poorly in cLV (Fig 9d–9h). Diffusion of fast green into the ISF also occurred. The distance of diffusion was about 1.5–2 mm away from the ventricle compartment.

## Discussion

Our results on 5HT and core-body temperature (*T*_cor_) confirmed previous reports indicating that intracranial administration of MDMA in a brain area could not cause serotonin syndrome [6–10]. It appears that intracranial administration of MDMA caused only a localized effect on extracellular 5HT at the delivery site, but did not affect the surrounding or distant nuclei necessary to process symptoms of serotonin syndrome. The cause of the localized effect was examined indirectly using chemical dye tracing. We provided a compelling evidence that chemical dyes by intracranial administration in one brain area poorly diffused into the ISF within the brain parenchyma, demonstrating that most other brain areas did not have any chemical dyes. MDMA diffusion following intracranial administration had patterns likely similar to chemical dyes. Therefore, we highlighted a new view explaining the lack of serotonin syndrome following intracranial MDMA administration. Specifically, a poor diffusion of MDMA from intracranial sites to the ISF within the brain parenchyma was more likely the mechanism responsible for the difference in MDMA-elicited serotonin syndrome compared to the systemic administration.

In the first set of studies, we compared five brain areas in response to systemic and intracranial administration of MDMA. Rationales for choosing those areas are two-fold. First, systemic administration of MDMA caused simultaneous and also excessive 5HT elevation in many brain areas [1,2], which may be the cause of serotonin syndrome. To determine whether intracranial administration could also cause such responses, it is necessary to investigate at least three different sites in the brain. The FCx, DEc and MRp, which are known to be involved in processing symptoms of serotonin syndrome [19–21], would be appropriate to be selected for this study. Second, the FCx, DEc and MRp represent the rostral, middle and caudal regions, respectively, in the brain. Thus, 5HT changes in three distinct areas are most likely to reflect what could happen across the entire brain, although a difference in symptom processing might exist between areas. In addition, analyzing the effects on the left hemisphere compared to the right hemisphere could provide valuable information on how the cerebroventricular system exerts distinct roles in drug distributions following intracranial administration. It has been recognized that MDMA toxicity can be time-dependent, displaying acute and delayed effects [1,25]. The acute effect due to an excess of extracellular 5HT arises immediately after administration and lasts in a few hours [1,26] resulting in a serotonin syndrome. There is a strong relationship between serotonin syndrome and *T*_cor_, demonstrating that serotonin syndrome can be characteristically discerned by measuring changes in *T*_cor_ [1,27]. In addition, when extracellular 5HT from all of these regions exceeded 10-fold above baseline, symptoms of serotonin syndrome may be elicited [5], suggesting that this level of 5HT is the threshold to elicit a serotonin syndrome. A secondary effect following serotonin syndrome is manifested by serotonergic neuronal injuries such as reduction in 5HT tissue contents and transporter (SERT) proteins [28–30] several days after administration. However, such molecular injuries are relatively rare unless high toxic doses (>7.5 mg/kg) are administered at warm temperatures [25]. The dose used in the present study was 2 mg/kg, which was relatively small and unlikely to elicit a delayed effect (unpublished observation). Therefore, studying molecular injuries by measuring changes in 5HT contents or SERT proteins might not be appropriate under our experimental conditions. Accordingly, measurements of changes in 5HT and *T*_cor_ were the most suitable approach to explore the role of the route of administration of MDMA on the development of serotonin syndrome.

One of the new findings in this study is that MDMA caused an administration route-dependent 5HT and *T*_cor_ responses. Intraperitoneal administration caused excessive 5HT elevation (>10 fold) in all sites investigated, as evident by measuring changes in the bilateral FCx and DEc and singular MRp. In addition, *T*_cor_ was reduced (hypothermia), suggesting the development of a mild serotonin syndrome [2,27]. Reverse microdialysis administration in the FCx, DEc or MRp also caused elevation in 5HT overflow exceeding the 10-fold threshold. However, there was no change in *T*_cor_, suggesting that reverse microdialysis administration of MDMA did not cause serotonin syndrome, consistent with previous reports [6–10]. One explanation for this is that the brain areas that regulate *T*_cor_, which can be affected by IP administration, are far away from the reverse microdialysis sites. To understand how far the reverse microdialysis exerts its effect, we implanted a second probe 2 mm away from the delivery sites. Interestingly, there were no detectable changes in 5HT, suggesting that reverse microdialysis can only cause a local effect, not beyond 2 mm from the delivery site.

This localized effect may be due to the property of microdialysis probes that have relatively a small exchange surface, e.g., 2.5 mm in length used for this study. The brain ventricles that have large surface areas can be used for drug delivery, being expected to cause a widespread effect on the CNS. Since early 1980s, ICV administration is considered to be an ideal option to deliver drugs prone to hepatic breakdown such as peptides and proteins [31–33]. Despite this, how drugs following ICV administration are distributed within the CSF in the four ventricles or whether there is diffusion to the ISF in other brain tissues has not been clearly investigated. Both the CSF and ISF arise from plasma, and eventually return into the circulation within a few hours of production [34,35]. One assumption widely accepted in literature is that there exists “free exchange” between the ISF and CSF, by which drugs injected into the CSF would freely diffuse into the ISF and then affect neurons in the parenchyma [31–33]. To test this hypothesis, we made a unilateral injection into the right LV while we measured 5HT simultaneously in five areas in the brain. We found that MDMA up to 100 μg produced excessive 5HT (i.e., >10-fold) only in the ipsilateral DEc and MRp, but not the contralateral DEc or FCx. This observation can be interpreted that MDMA administered by the intracranial route can reach some but not all brain sites investigated. Findings that ICV administration had no effect on *T*_cor_ support the hypothesis that the brain areas critical for regulating *T*_cor_ could not be reached from this route of administration.

Could the observation be interpreted as an action of metabolites but not MDMA itself as suggested previously [6,10]? To answer this question, we conducted reverse microdialysis administration demonstrating that MDMA at 10 μM (not corrected from a probe delivery rate) produced excessive 5HT elevation in those brain sites, suggesting that those brain sites were sensitive to MDMA. Thus, MDMA itself could not be excluded from the list of psychoactive drugs, and an alternative hypothesis should be tested. One possibility is that brain areas that are distant away from the injection site may not be reachable by ICV administration. Thus, drug concentration in a given area depends on a distance to the injection site. This hypothesis can be theoretically tested by measuring MDMA levels at different brain sites using chromatographic-mass spectrometric analysis [36]. However, this equipment was not available in our laboratory. Instead, we utilized chemical dyes as an indirect approach that might provide some clues. Fast green and DiI are two chemical dyes with distinct hydrophilic and lipophilic properties, respectively, which are commonly used to locate the placement of injectors in the brain [37,38]. To minimize the amount of chemicals used in rats, dyes in some tests were administered by an IV route. That change would not have an impact on quality of data comparison because our purpose was to determine how dyes were distributed in the ISF of brain parenchyma following systemic administration. Nevertheless, we traced the dye distribution and diffusion patterns within the brain following systemic, reverse microdialysis or ICV administration. After systemic administration, dyes can be transported from the injection site to brain capillaries that are widely distributed in the brain. Furthermore, since each neuron receives supports from several capillaries [35], the dye distribution must be very efficient. After distribution, diffusion takes place instantaneously from capillary blood to the ISF cross the vascular barriers. Consistent the expectation, we found that DiI fluorescence was evenly distributed in the entire brain when administered systemically.

Following ICV administration, however, there was no DiI fluorescent trace found in the parenchymal tissues such as the FCx (Fig 6). The observation is consistent with results obtained from MDMA investigation, suggesting the brain areas affected by systemic administration may not be reachable by the ICV administration. Although DiI fluorescence was not detectable in the FCx, it was seen in the ventricles, arguing against possible involvement of dye metabolites. Taken together, the use of fluorescent dyes in this study could overcome the technique limitation in understanding the differential results between systemic and intracranial administration.

We showed that dyes were not within capillaries, suggesting that ICV administration cannot utilize the brain capillary system to distribute dyes from the injection site to other sites. Dye transfer took place in the CSF. In the imaging study, we found that chemical dyes move from the ipsilateral LV to the 3V and the 4V, which appears to follow the natural flow of CSF (see reviews; [35]). This implicates that drugs administered in the LV travels more favorably in the CSF towards the 3V and 4V rather than diffuse through the ependymal cell lining of the ventricles into the ISF. For the following discussion, the term “distribution” is arbitrarily defined as spreading of drugs in the four ventricle compartments while “diffusion” as the movement toward the ISF compartment. It is known that CSF flows in the four ventricles at the rate of 2 μl/min [35]. For this reason, the rate of ICV infusion in this study was set at 0.2 μl/min, much lower than the CSF flow rate, to ensure that the drug could be taken into the CSF and not flood out of the guide cannula, or increase cranial pressure that would cause animal discomfort. Because of the characteristic direction of CSF flow, our DiI data (Fig 8) corresponded with the fast green results (Fig 9) demonstrating that chemicals delivered into the right LV would follow the natural flow in the CSF and distribute mainly in the ipsilateral LV, 3V and 4V. We also observed that there was the chemical dyes in the contralateral LV, but relatively small. This might be due to animal head movement, gravity, and respiration or arterial impulse that affects the CSF flow [39]. The difference in dye distribution between the two LV compartments supported our neurochemical findings that 5HT response was more pronounced in the ipsilateral DEc compared to the contralateral DEc. Since the MRp is closely located near the path of CSF flow (see Fig 9), MDMA could exert more effects on 5HT in this site than the FCx or contralateral DEc, which may well explain the different responses among the three brain sites (Fig 4).

One may wonder whether increasing lipid solubility facilitates drug diffusion from the CSF to ISF. To test such possibility, we compared the diffusion pattern of the hydrophilic dye (i.e., fast green, Fig 9) with the lipophilic dye DiI (Fig 8). Results showed no difference between two dyes in diffusion from the ventricles to the ISF. Similarly, MDMA lipophilicity could not be the factor for increasing diffusion into the ISF. Since diffusion is a passive event that depends on a concentration gradient, whether the drug dose in the ventricles was enough to build up the high concentration gradient relative to the ISF could be an important question. In this regard, we examined several different doses up to 100 μg MDMA. Given that the LV volume in the brain is only ~43 μL [40], 100 μg could theoretically generate a concentration as high as 10 mM (or 10,000 μM). On the other hand, a low micromolar level (<10 μM) of MDMA in the ISF is sufficient to cause excessive 5HT over 10-fold above baseline ([41–43], also in this study). This suggests that the MDMA concentration in the CSF was at least 1000 times greater than that needed in the ISF. Therefore, the concentration gradients are unlikely the factor preventing MDMA in the CSF from widespread diffusion into the ISF. Rather, MDMA in the CSF cannot freely diffuse into the ISF as expected previously [44,45].

In summary, our results are consistent with previous observations demonstrating that systemic but not intracranial MDMA produces the serotonin syndrome in animals [6–10]. For many years, the different responses between systemic and intracranial administration have been ascribed almost exclusively to drug hepatic metabolites [11,16,46]. In the present study, we demonstrated that MDMA following intracranial administration could not reach the brain areas activated to elicit the serotonin syndrome. A limitation of this study was that we had no direct approach to measure the MDMA levels in those brain areas. To overcome the limitation, chemical dyes were employed to investigate indirectly the relationship between drug administration and diffusion. We found that dyes administered in one brain site poorly diffused into the ISF of other brain sites. Thus the study showed that one additional pivotal consideration to explain the lack of serotonin syndrome following intracranial administration is the potential for poor diffusion of MDMA from injection sites to critical areas of the brain.
